# Hepatotoxicity Due to Azole Antimycotic Agents in a HLA B*35:02-Positive Patient

**DOI:** 10.3389/fphar.2019.00645

**Published:** 2019-06-11

**Authors:** Tim Bühler, Michael Medinger, Jamal Bouitbir, Stephan Krähenbühl, Anne Leuppi-Taegtmeyer

**Affiliations:** ^1^Department of Clinical Pharmacology and Toxicology, University Hospital Basel, Basel, Switzerland; ^2^Divisions of Hematology and Internal Medicine, Department of Medicine, University Hospital Basel, Basel, Switzerland

**Keywords:** azole antifungal agents, voriconazole, posaconazole, isavuconazole, hepatotoxicity, drug-induced liver injury (DILI), cross-toxicity, HLA B*35:02

## Abstract

We will present a 42-year-old woman with acute myeloid leukemia and pulmonary aspergillosis. She was treated with several antifungal agents, including three triazoles. Voriconazole, posaconazole, and isavuconazole all led to hepatocellular liver injury. Voriconazole administration led to a peak alanine aminotransferase (ALT) value of 1,793 U/L (normal range, 9–59 U/L). After posaconazole and isavuconazole treatment, ALT rose over 500 U/L. The typical course of events, exclusion of differential diagnoses, and normalization of the liver function tests (LFTs) after stopping the triazoles were highly suspicious for a drug-induced liver injury (DILI). Interestingly, our patient carries a rare HLA B allele (HLA B*35:02), which occurs in less than 1% of the population and is known to be associated with minocycline-induced liver injury. Over the course of 4 months, the patient received two induction chemotherapies and afterward underwent a successful allogenic hematopoietic stem cell transplantation. Her liver function recovered rapidly and favorable clinical findings concerning the aspergillosis led to a de-escalation of the antifungal treatment to prophylactic dose fluconazole. Delayed hepatotoxicity suggested a dose dependency and a cumulative effect. The question of a common pathophysiology and a cross-toxicity was raised. At the present time, only a few case reports describe cross-toxicity or its absence after rechallenge with different azoles. The pathophysiology is not well understood. Ketoconazole was found to impair rat mitochondrial function *in vitro*. Further investigations showed cell membrane toxicity and ATP depletion in isolated human liver cancer cells. Our case report suggests a cross-toxicity, dose-dependency, and a possible genetic predisposition of triazole-induced liver injury.

## Background

Azole antifungal agents are among some of the most widely used antifungal drugs. Their mechanism of action is inhibition of the fungal synthesis of ergosterol by inhibiting lanosterol 14α-demethylase, a cytochrome P450 enzyme (Kyriakidis et al., 2017). The inhibition of this major sterol component disturbs the integrity of the fungal plasma membrane (Boucher et al., 2004). Two groups make up the clinically useful antifungals: the imidazoles (for example, ketoconazole and clotrimazole), which are mainly used in the treatment of superficial mycoses, and the triazoles (for example, itraconazole, voriconazole, and posaconazole), which are more specific for fungal than for mammalian P450 enzymes. Triazoles, therefore, have a broader clinical application in both the treatment of superficial and systemic fungal infections (Groll et al., 2003). The triazoles are divided into first-generation substances (fluconazole, itraconazole) and second-generation substances (voriconazole, posaconazole, isavuconazole). Voriconazole and posaconazole have an extended spectrum of activity against yeasts, *C. neoformans*, and molds (Chen and Sorrell, 2007). In general, antifungal treatment is individualized to the patient’s clinical and radiological response. The treatment of invasive pulmonary aspergillosis is often prolonged, lasting from several months to more than 1 year (Limper et al., 2011; Corzo-Leon et al., 2015). Therefore, patients are exposed to a high cumulative dose of azoles.

Today, drug-induced liver injury (DILI) is one of the commonest reasons for the withdrawal of an approved drug from the market. It is, therefore, an important issue during drug development and clinical application (Navarro and Senior, 2006). We present the case of a 42-year-old woman with newly diagnosed acute myeloid leukemia (AML). During the first induction chemotherapy, the patient developed pulmonary aspergillosis, which was treated with multiple antifungal agents. The patient gave written consent for publication of her case.

## Case Presentation

**Figure 1:**
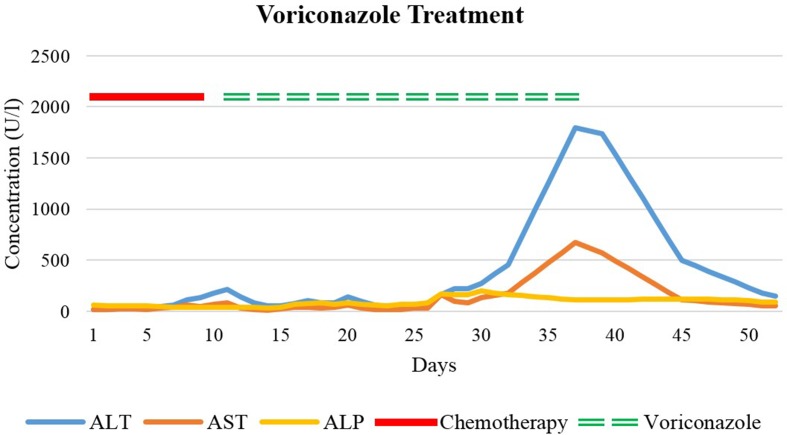
Liver function test values before, during, and after treatment with voriconazole. Day 1 refers to the first day of the first induction chemotherapy.

Our patient is a 42-year-old woman with AML diagnosed in August 2018 after 3 months of non-specific symptoms, including recurrent herpes infections, headaches, and fatigue. Her first induction chemotherapy was with idarubicin, an anthracycline, and cytarabine (a synthetic pyrimidine analogue). After several days of therapy, she developed neutropenia and severe diarrhea due to mucositis, leading to hypophosphatemia and hypokalemia. The patient was treated for fever in neutropenia initially with cefepime and amikacin and then with piperacillinum/tazobactam due to a presumed allergic reaction to cefepime or amikacin. Because of persistent fever, piperacillinum/tazobactam was switched to meropenem, and because of the severe mucositis, caspofungin was added. Persisting symptoms and rising inflammatory parameters over the next days led to further investigations. On chest CT scan, five small pulmonary nodules (measuring less than 4 mm) were detected. This finding together with the associated febrile neutropenia led to the suspicion of pulmonary aspergillosis. Her antifungal therapy was switched from caspofungin to intravenous, and then to oral voriconazole. Voriconazole trough concentration measurements were within the therapeutic range (1–6 mg/L). Under treatment with additional broad-spectrum antibiotics (meropenem, aztreonam, and vancomycin due to multiple bacterial infections), she showed a good clinical response. Ten days after starting voriconazole, however, her liver transaminases rose, accompanied by only slightly elevated cholestatic parameters and normal bilirubin levels (**Figure 1**). After 3 weeks of voriconazole therapy, alanine aminotransferase (ALT) (reference range, 8–41 U/L) reached its peak value of 1793 U/L, and antifungal therapy was terminated. Aspartate aminotransferase (AST) (reference range, 11–34 U/L) reached a peak value of 672 U/L on the same day. Alkaline phosphatase (ALP) (reference range, 35–105 U/L) values oscillated from 80 U/L up to nearly 200 U/L, reaching a peak value of 197 U/L after about 2 weeks of treatment. Cessation of the triazole administration led to a normalization of the LFTs. Due to their different half-lives in plasma, AST normalized first, followed by ALT, and then the cholestatic parameters.

**Figure 2:**
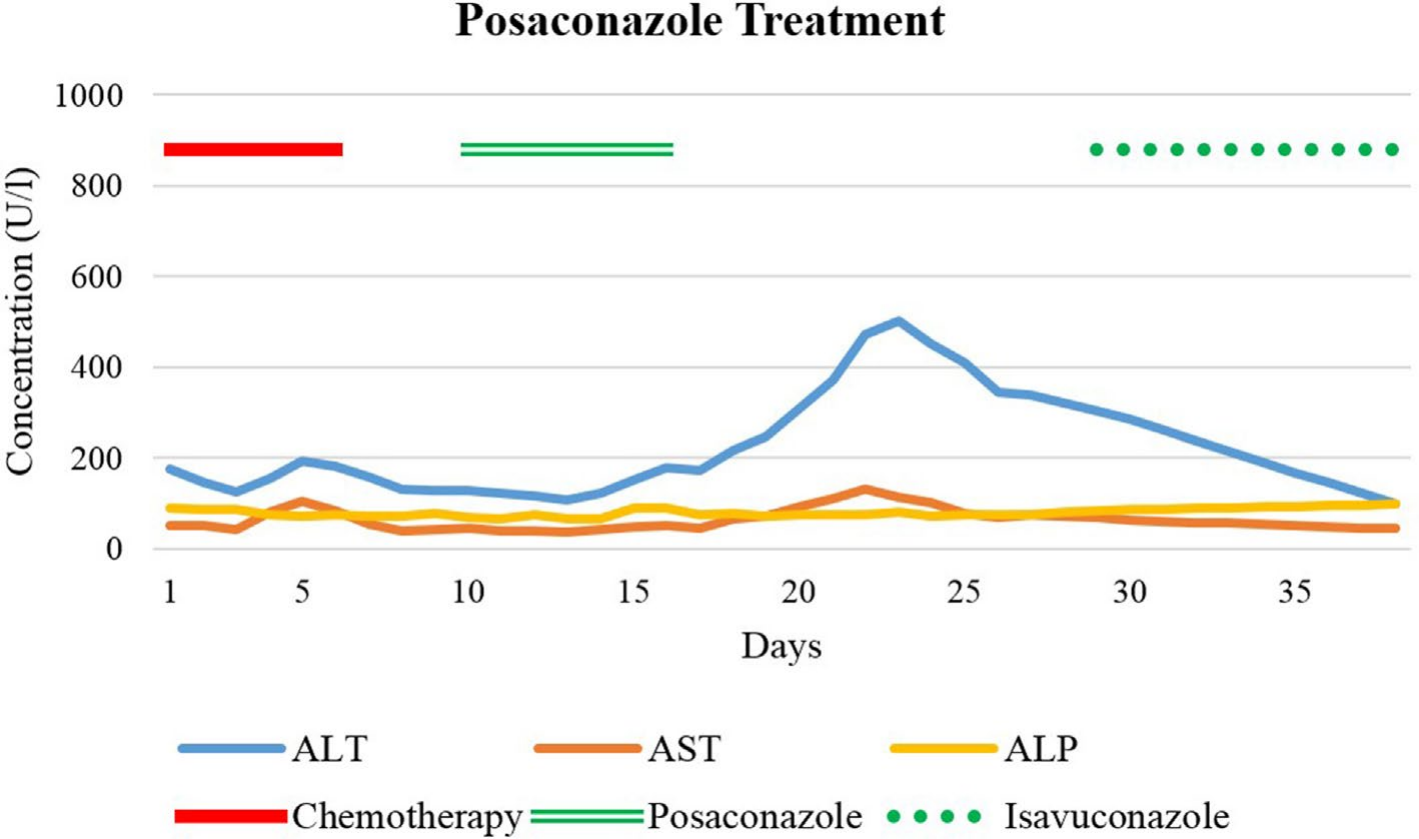
Liver function test values before, during, and after treatment with posaconazole. Day 1 refers to the first day of the second induction chemotherapy. Isavuconazole treatment was started on day 29.

Six weeks later, the patient received the second course of induction chemotherapy. After several days of treatment with the nucleoside analog cytarabine and amsacrine, a synthetic topoisomerase II inhibitor, the patient again developed severe diarrhea and fever in neutropenia. Two sets of blood cultures grew *Streptococcus mitis*. Broad-spectrum antibiotics were commenced (cefepime, ceftriaxone, and piperacillin/tazobactam). Caspofungin was administered to treat the suspected pulmonary aspergillosis. After 1 week of caspofungin treatment and stable LFTs despite chemotherapy, antifungal therapy was switched to oral posaconazole tablets for better coverage of mucor species. On the first day, 300-mg oral posaconazole was administered twice daily as a loading dose, followed by 300 mg daily for 5 days. A low posaconazole blood concentration on treatment day 4 (0.5 mg/L; target range, 1.26–3.74 mg/L) prompted a dose increase to 400 mg once daily on day 6. After 4 days of posaconazole treatment, liver enzymes began to rise (**Figure 2**).

Posaconazole was discontinued and therapy switched to caspofungin again. However, despite this, transaminases continued to rise (**Figure 2**). Our department of clinical pharmacology and toxicology was consulted and asked to investigate the cause of the rising liver enzymes and to make suggestions for the further management. ALT was 179 U/L at that time, whereas ALP and bilirubin remained normal. We systematically worked up all drugs administered over the past months, evaluated their potential hepatotoxicity [LiverTox.NIH.gov (internet). Bethesda: Food and Drug Administration; c2019 (cited 2019 Mar 01). Available from: https://www.livertox.nih.gov], determined temporal associations, and assessed differential diagnoses. Therapeutic drug monitoring (TDM) was performed three times for voriconazole and once for posaconazole. All serum concentrations were within the therapeutic range. Abdominal CT scans revealed no signs of liver or portal vein thrombosis, masses, hepatic steatosis, or biliary obstructions. Serology showed no signs of viral infections, and eosinophils were within normal limits on complete blood count, incongruous with the diagnosis of “drug reaction with eosinophilia and systemic symptoms” (DRESS) (Walsh and Creamer, 2011) but difficult to interpret due to aplasia under chemotherapy. On account of the low thrombocyte count, a liver biopsy was not performed. Altogether, we concluded that voriconazole and posaconazole most likely caused both episodes of liver enzyme elevation.

**Figure 3:**
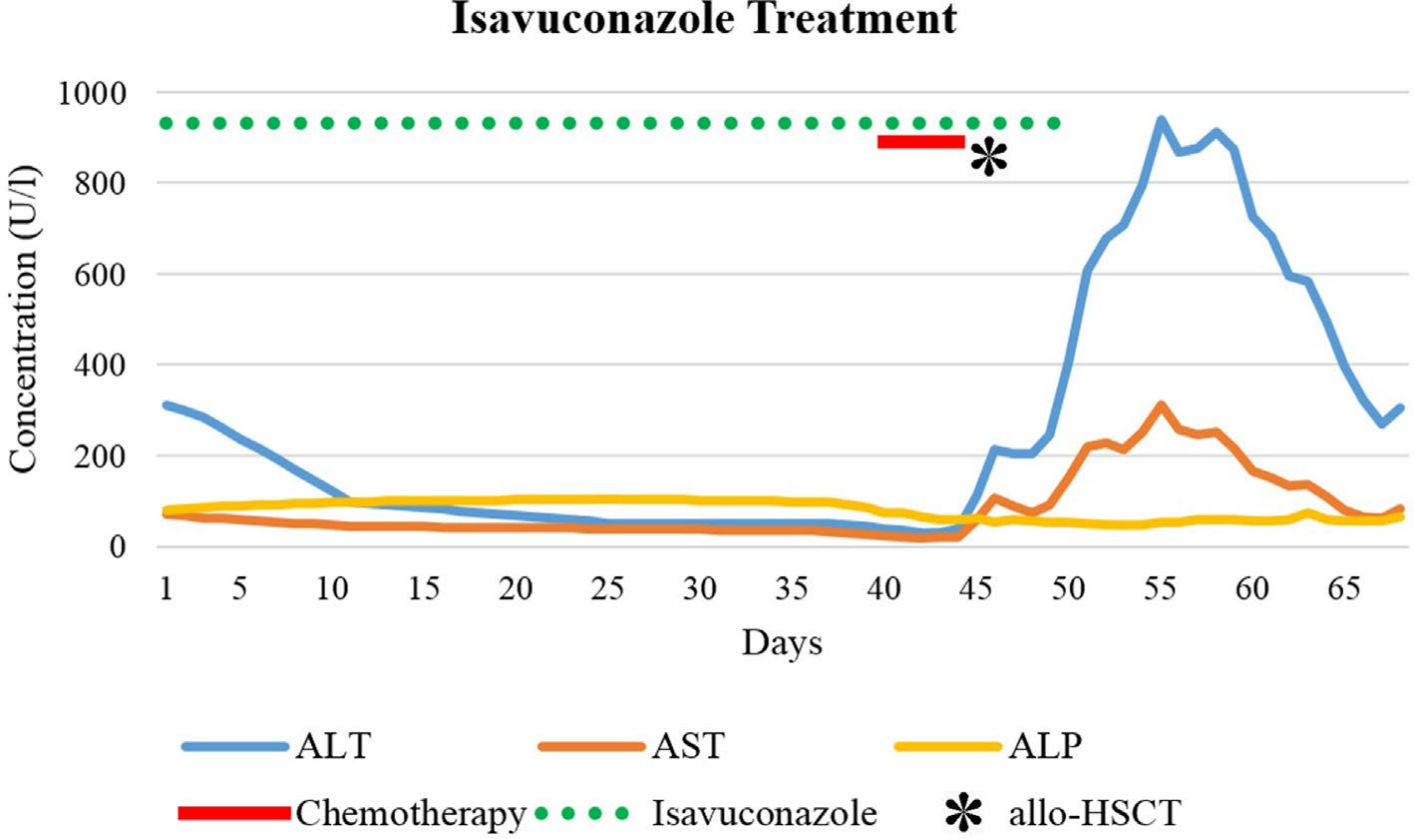
Isavuconazole treatment, chemotherapy, and allogenic HSCT (day 46). Day 1 refers to the first day of isavuconazole treatment.

AST reached its peak value of 130 U/L 6 days after the last posaconazole administration followed by ALT (peak value of 502 U/l) on the following day. Gamma-glutamyltransferase (GGT) (reference range, 6–40 U/L) was only slightly increased (97 U/L); ALP and bilirubin again remained within normal limits. Over the next days, LFTs returned to baseline and the patient responded clinically too. Chest CT scan was repeated before discharge. Over the preceding 2 months, the five pulmonary nodules had increased considerably in size, strengthening the suspicion of pulmonary aspergillosis. Our infectious diseases specialists recommended one of two remaining treatment options: continuation of caspofungin, given intravenously once daily, or initiation of isavuconazole (Cresemba^®^) 200 mg once daily orally. Unfortunately, several organizational and actuarial reasons made once daily infusions after discharge impossible. The patient was informed about the risk of hepatotoxicity with isavuconazole, possibly resulting in severe and dangerous liver damage. Nevertheless, after burdensome therapies and many weeks of hospital stay, she decided to take isavuconazole and repeatedly have her LFTs monitored. During the first weeks of treatment with isavuconazole, transaminases steadily decreased, and the patient improved clinically (**Figure 3**). After 5 weeks of continuous treatment, 2 days before the planned allogenic hematopoietic stem cell transplantation (allo-HSCT), ALT and AST began to rise again. Seven weeks after successful isavuconazole treatment, the drug had to be stopped. Transaminases rose for another 1.5 weeks before returning to baseline, similar to posaconazole.

The allo-HSCT was successful. Interestingly, our patient as well as her donor—a blood relative—were both carriers of a rare HLA class I antigen (B*35:02, found in only 0.912% of the Caucasian population) [ZKRD.de/ (internet). Ulm: The ZKRD. The German National Bone Marrow Donor Registry [Internet]. Ulm: The ZKRD. 1992 - [cited 2019 Mar 4]. Available from: https://www.zkrd.de/. The patient recovered rapidly after the allo-HSCT. Favorable clinical findings concerning the aspergillosis led to a de-escalation of the antifungal treatment to prophylactic dose fluconazole (400 mg orally once weekly). We reported the adverse drug reactions—DILI under voriconazole, then posaconazole, and then isavuconazole—to the national drug authority’s department of pharmacovigilance (Swissmedic).

## Discussion

We describe a case of a 42-year-old woman with a history of AML and pulmonary aspergillosis who developed DILI after treatment with three different azole antimycotics. To our knowledge, there are no other case reports of hepatotoxicity recurring in the same individual treated with three different azoles. Idiosyncratic DILI is a rare adverse hepatic reaction occurring under therapeutic dosages that is unexpected on the basis of the pharmacological action of the drug administered (Aithal et al., 2011). These reactions are, therefore, different from DILI secondary to drug overdose (e.g., acetaminophen). Despite advances in diagnostics, today, DILI still remains a diagnosis of exclusion. In general, we follow the consensus definition of DILI recommended in Aithal et al. (2011). These include any of the following: 1) more than or equal to 5× elevation above the ULN for ALT, 2) more than or equal to 2× elevation above the ULN for ALP, and 3) more than or equal to 3× elevation in ALT concentration and simultaneous elevation of bilirubin concentration more than 2× the ULN. Elevations of 5′-nucleotidase and/or GGT often accompany cholestatic patterns, providing additional information about the damage location. The R quotient (R = (ALT/ULN)/(ALP/ULN); ULN = upper limit of normal) defines whether the pattern of the injury is hepatocellular (R ≥ 5), cholestatic (R ≤ 2), or mixed (2 < R < 5). Bilirubin elevations and jaundice without biliary obstruction indicate a poor prognosis of the DILI (referred to as Hy’s law) (Reuben, 2004; Bjornsson, 2006). Overall, approximately 3% of DILI cases are caused by antimycotic agents (Raschi et al., 2014; Doss et al., 2017). A broad spectrum of hepatotoxic reactions to triazoles has been described. Mild and transient elevations of transaminases, cholestasis, hepatitis, and even cases of liver failure have occurred. In general, the use of triazoles can lead to both hepatocellular, as well as cholestatic patterns of liver damage (Zimmerman, 1999). Pharmacokinetics of the different drugs are essential in predicting the rise and fall of LFTs.

Oral voriconazole (Vfend^®^) has a high bioavailability, is extensively distributed into tissue, and is primarily metabolized by the liver (e.g., CYP3A4, CYP2C9, CYP2C19) (Boucher et al., 2004; Food and Drug Administration, 2010). Pharmacokinetics are nonlinear, leading to a dose-dependent terminal half-life in adults (Purkins et al., 2002). Studies using radioactively labeled voriconazole demonstrated that most of the drug was eliminated during the first 96 h after i.v. and oral dosing (Food and Drug Administration, 2010). In our patient, DILI criteria were observed after 10 days of voriconazole treatment. ALT and ALP were both increased to a similar degree (R approximately 3.5), indicating a mixed pattern of damage. To predict the sequence of LFT normalization after treatment discontinuation, half-lives of the suspected causative drug and half-lives of individual LFTs must be considered. AST has a plasma half-life of about 17 h, ALT about 47 h, whereas ALP has a plasma half-life up to 7 days (Dufour et al., 2000a; Dufour et al., 2000b; Giannini et al., 2005). After treatment discontinuation in our case, AST decreased initially, followed by ALT and ALP. Posaconazole (Noxafil^®^) absorption is saturable when given orally (Ezzet et al., 2002). Due to the long half-life (25–31 h), posaconazole stays in the body for 5 to 10 days after discontinuation (Food and Drug Administration, 2015b). In our patient, despite discontinuation of posaconazole, LFTs increased steadily for another week, before reaching their peak level and then declining, in keeping with posaconazole’s long half-life prolonging the hepatotoxicity (Food and Drug Administration, 2015b). Our case showed a hepatocellular pattern (R approximately 7.4). Isavuconazole (Cresemba^®^) was approved by the FDA in 2015 and has shown to be a non-inferior alternative to voriconazole for the treatment of invasive mold disease with even fewer adverse effects (Food and Drug Administration, 2015a; Maertens et al., 2016). Of particular note is its long mean plasma half-life (130 h). Delayed hepatotoxicity suggests that cumulative drug exposure might be relevant, and we suggest that chemotherapy followed by allogenic hematopoietic stem cell transplantation might have contributed to the reaction. Nowadays, TDM is widely recognized as a valuable tool to improve safety and efficacy of antifungal treatment. Of note are the long half-lives of many azoles, which leads to a delayed steady-state. Within the group of azoles, TDM should be performed in the majority of patients receiving treatment with voriconazole and posaconazole (Ashbee et al., 2014). The necessity of TDM for posaconazole given in prophylactic doses is debatable (Tverdek et al., 2017). Furthermore, target concentrations for treatment doses for posaconazole are not as well established as for other azoles. On the one hand, higher drug exposures were associated with a higher clinical response in patients with invasive aspergillosis (Walsh et al., 2007). On the other hand, there are concerns about an increased risk of hepatotoxicity with higher posaconazole doses (Tverdek et al., 2017). In contrast, TDM of isavuconazole is not yet routinely performed (Van Matre et al., 2019), but methods for routine TDM such as high-performance liquid chromatography assays are current research object (Mueller et al., 2018).

A genetic predisposition appears to play a role in the development of DILI and its clinical pattern (Kaliyaperumal et al., 2018). Genetic variations predisposing to the development of azole-induced hepatotoxicity are—to the best of our knowledge—however, not yet known (Kullak-Ublick et al., 2017; Kaliyaperumal et al., 2018). Interestingly, our patient is a carrier of HLA B*35:02—a rare allele found in only 0.912% of the Caucasian population [ZKRD.de/ (internet). Ulm: The ZKRD. The German National Bone Marrow Donor Registry; c2019 (cited 2019 Mar 4). Available from: https://www.zkrd.de/]. Ulm: The ZKRD. The German National Bone Marrow Donor Registry; c2019 [ZKRD.de/ (internet). Ulm: The ZKRD. The German National Bone Marrow Donor Registry; c2019 (cited 2019 Mar 4). Available from: https://www.zkrd.de/]. Available from: https://www.zkrd.de/, #8. This rare HLA B*35:02 allele is known to be associated with an increased risk of DILI after minocycline treatment (Urban et al., 2017). The mechanism is not well understood. Minocycline and its metabolites might act as haptens, which might either bind to cellular peptides and proteins or directly to the HLA B*35:02. The peptides are then presented on the major histocompatibility complex (MHC) class I molecules on the surface of the cells. Immune cells, e.g., CD8^+^ cytotoxic T-lymphocytes, recognize the altered receptors and trigger an immune response. This concept was described in flucloxacillin-associated DILI and HLA B*57:01 carriers: The drug-peptide complex has led to the activation of T cells (Monshi et al., 2013). Other discussed mechanisms for the hepatotoxic properties of minocycline include the formation of neoantigens by damaged hepatocytes (Urban et al., 2017), as well as an increased production of reactive intermediates (Mannargudi et al., 2009). It is interesting to note that the HLA B*57:01 allele is not only associated with flucloxacillin-induced liver injury, but also pazopanib-induced liver injury and abacavir hypersensitivity, suggesting that HLA allele associations are not specific for a single drug.

The risk of liver toxicity of different azoles was compared in a cohort study between 2004 and 2010 (Lo Re et al., 2016). Liver aminotransferases > 200 U/L (ALT or AST) occurred more commonly after treatment with voriconazole (181.9/1,000 person-years) and posaconazole (191.1), compared with fluconazole (13.0), ketoconazole (19.3), and itraconazole (24.5). Severe acute liver injury events occurred at a median time from azole initiation of 22 days (interquartile range, between 9 and 32 days). They were uncommon with fluconazole (2.0/1,000 person-years), ketoconazole (2.9), and itraconazole (0.0), but an issue with voriconazole (16.7) and posaconazole (93.4) (Lo Re et al., 2016). Isavuconazole was not yet available and therefore not included. To date, the mechanism of hepatotoxicity associated with azoles is not entirely clear. An early study showed that ketoconazole impaired mitochondrial function in isolated rat liver mitochondria (Rodriguez and Acosta, 1996). Later, *in vitro* investigations performed in HepG2 (human liver cancer cell line) and HepaRG (differentiated human hepatocellular carcinoma cells) showed that ketoconazole and posaconazole were associated with cell membrane toxicity and ATP depletion (Haegler et al., 2017). Moreover, the exposure to both drugs in HepG2 cells showed the dissipation of the mitochondrial membrane potential and impaired activity of enzyme complexes of the mitochondrial electron transport chain. Consequently, these impairments increased the production of mitochondrial reactive oxygen species (ROS), leading to the formation of mitochondrial oxidative stress. The formation of oxidative stress then provoked the activation of the mitochondrial pathway of apoptosis. Interestingly, we showed that the presence of a pre-existing defect in mitochondrial function of HepG2 cells is a susceptibility factor that can trigger cellular toxicity at concentrations which are not toxic when this defect is absent. Unlike ketoconazole and posaconazole, which we showed to be mitochondrial toxicants, fluconazole and voriconazole were not toxic in our models (Haegler et al., 2017). Other cases of azole-induced liver injury found no recurrence on switching azoles (Spellberg et al., 2003; Foo and Gottlieb, 2007). The case reported by Foo and Gottlieb had a predominant cholestatic DILI, which points to a different pathophysiological mechanism, compared to our patient (Foo and Gottlieb, 2007). The different mechanisms of DILIs are complex and currently the subject of investigation. Among cholestatic patterns, inhibition of bile salt export pumps like the multidrug resistance protein 3 seems to be a key element (Stieger and Mahdi, 2017). Other possible mechanisms of drug-related hepatotoxicity include “damage-associated molecular patterns” (DAMPs) (Kato and Uetrecht, 2017), cytokines, such as TNF-α or IFN-γ (Roth et al., 2017), drug-specific T-cells (Ogese et al., 2017), and mitochondrial dysfunction (Hu et al., 2016). The rare HLA allele might point to an association with genetic factors.

In conclusion, in our patient, the typical course of events, known drug toxicities and similar patterns of liver enzyme elevation of the three DILI episodes led to our conclusion of a class effect of azole hepatotoxicity. We suggest that the chemotherapy increased the patient’s susceptibility to liver injury during treatment with the different azoles. This led to unexpected temporal relationships between drug exposure and DILI development, which initially made pharmacovigilance assessment challenging. However in all three episodes, drug dechallenge led to a recovery of LFTs in keeping with the known drug and enzyme half-lives in each case. The pathophysiology of DILI remains an object of ongoing investigation. An increasing list of adverse reactions can be linked to specific HLA alleles. Efforts in postmarketing surveillance and reporting of cases to public health authorities are crucial due to the ongoing approval of new drugs and unknown adverse effects of widely used drugs and herbs (Real et al., 2019). In our opinion, a better understanding of basic pathophysiology, genetics, and immunology is mandatory for future prevention and management of DILI.

## Data Availability Statement

The raw data supporting the conclusions of this manuscript will be made available by the authors, without undue reservation, to any qualified researcher.

## Ethics Statement

The patient gave written informed consent for the publication of her case.

## Author Contributions

TB and AL-T conceived, designed, and wrote the first draft of the case report. MM—the patient’s attending physician—provided critical information regarding the clinical course and the specific treatments. JB revised the work and included crucial information about the pathophysiology of the stated condition. SK contributed to the interpretation of the clinical data and revised the work.

## Conflict of Interest Statement

The authors declare that the research was conducted in the absence of any commercial or financial relationships that could be construed as a potential conflict of interest.
